# Supplementary material for: Venous Resection During Pancreatoduodenectomy for Pancreatic Ductal Adenocarcinoma—A Multicentre Propensity Score Matching Analysis of the Recurrence After Whipple’s (RAW) Study
Source: Cancers (Basel). 2025 Apr 4;17(7):1223. doi: 10.3390/cancers17071223 (PMC11987722; doi:10.3390/cancers17071223)
Supplement: Supplementary file 1 [file cancers-17-01223-s001.zip › supplementary-material.pdf]

Original Article

Oncological Impact of Pancreatoduodenectomy with concomitant Venous Resection for Pancreatic Ductal Adenocarcinoma – a Multicentric Propensity Score Matching from the Recurrence after Whipple's (RAW) study

Ruben Bellotti[1], Florian Ponholzer[1], Benno Cardini[1], Thomas B. Russel[2], Peter L. Labib[2], Stefan Schneeberger[1], Fabio Ausania[3], Elizabeth Pando[4], Keith J. Roberts[5], Ambareen Kausar[6], Vasileios K. Mavroeidis[7,8], Gabriele Marangoni[9], Sarah C. Thomasset[10], Adam E. Frampton[11], Pavlos Lykoudis[12], Nassir Alhaboob[13], Hassaan Bari[14], Andrew M. Smith[15], Duncan Spalding[16], Parthi Srinivasan[17], Brian R. Davidson[18], Ricky H. Bhogal[8], Daniel Croagh[19], Ismael Dominguez[20], Rohan Thakkar[21], Dhanny Gomez[22], Michael A. Silva[23], Pierfrancesco Lapolla[24], Andrea Mingoli[24], Alberto Porcu[25], Nehal S. Shah[26], Zaed Z. R. Hamady[27], Bilal Al-Sarrieh[28], Alejandro Serrablo[29], Somaiah Aroori[2]; RAW Study Collaborators; Manuel Maglione[1]

*Correspondence: manuel.maglione@i-med.ac.at; Anichstr. 35, 6020 Innsbruck, Tirol (AT);

Tel.: +43-512-504-80809; FAX: 43-512-504-22602

Affiliations
1. Department of HPB Surgery, Medical University of Innsbruck, Innsbruck, Austria.
2. Department of HPB Surgery, University Hospitals Plymouth NHS Trust, Plymouth, UK.
3. Department of HPB Surgery, Hospital Clínic de Barcelona, Barcelona, Spain.
4. Department of HPB Surgery, Hospital Universitari Vall d'Hebron, Barcelona, Spain.
5. Department of HPB Surgery, University Hospitals Birmingham NHS Foundation Trust, Birmingham, UK.
6. Department of HPB Surgery, East Lancashire Hospitals NHS Trust, Blackburn, UK.
7. Department of HPB Surgery, University Hospitals Bristol and Weston NHS Foundation Trust, Bristol, UK.
8. Department of HPB Surgery, The Royal Marsden NHS Foundation Trust, London, UK.
9. Department of HPB Surgery, University Hospital Coventry & Warwickshire, Coventry, UK.
10. Department of HPB Surgery, NHS Lothian, Edinburgh, UK.
11. Department of HPB Surgery, Royal Surrey NHS Foundation Trust, Guildford, UK.
12. Department of HPB Surgery, Hull University Teaching Hospitals NHS Trust, Hull, UK.
13. Department of HPB Surgery, Ibn Sina Specialized Hospital, Khartoum, Sudan.
14. Department of HPB Surgery, Shaukat Khanum Memorial Cancer Hospital, Lahore, Pakistan.
15. Department of HPB Surgery, Leeds Teaching Hospitals NHS Trust, Leeds, UK.
16. Department of HPB Surgery, Imperial College Healthcare NHS Trust, London, UK.
17. Department of HPB Surgery, King's College Hospital NHS Foundation Trust, London, UK.
18. Department of HPB Surgery, Royal Free London NHS Foundation Trust, London, UK.
19. Department of HPB Surgery, Monash Medical Centre, Melbourne, Australia.
20. Department of HPB Surgery, Salvador Zubiran National Institute of Health Sciences and Nutrition, Mexico City, Mexico.
21. Department of HPB Surgery, Newcastle upon Tyne Hospitals NHS Foundation Trust, Newcastle upon Tyne, UK.
22. Department of HPB Surgery, Nottingham University Hospitals NHS Trust, Nottingham, UK.
23. Department of HPB Surgery, Oxford University Hospitals NHS Foundation Trust, Oxford, UK.
24. Department of HPB Surgery, Policlinico Umberto I University Hospital Sapienza, Rome, Italy.
25. Department of HPB Surgery, Azienda Ospedaliero Universitaria di Sassari, Sassari, Italy.

26. Department of HPB Surgery, Sheffield Teaching Hospitals NHS Foundation Trust, Sheffield, UK.
27. Department of HPB Surgery, University Hospital Southampton NHS Foundation Trust, Southampton, UK.
28. Department of HPB Surgery, Swansea Bay University Health Board, Swansea, UK.
29. Department of HPB Surgery, Hospital Universitario Miguel Servet, Zaragoza, Spain.

Collaborators

RAW Study Collaborators:

Somaiah Aroori, Peter L Labib, Thomas B Russell, Adam Streeter, Jemimah Denson, David Sheridan, Mark Puckett, Matthew G Browning, Fabio Ausania, Carolina Gonzalez-Abos, Elizabeth Pando, Nair Fernandes, Elsa Garcia Moller, Cristina Dopazo Taboada, Keith J Roberts, Rupaly Pande, Jameel Alfarah, Ambareen Kausar, Samik Bandyopadhyay, Ahmed Abdelrahim, Ayesha Khan, Vasileios K Mavroeidis, Caitlin Jordan, Jonathan R E Rees, Gabriele Marangoni, Harry Blege, Sarah C Thomasset, William Cambridge, Olga White, Adam E Frampton, Sarah Blacker, Jessie Blackburn, Casie Sweeney, Pavlos Lykoudis, Daniel Field, Mohammed Gouda, Manuel Maglione, Ruben Bellotti, Nassir Alhaboob, Hytham K S Hamid, Hassaan Bari, Hassan Ahmed, Andrew M Smith, Catherine Moriarty, Louise White, Mark Priestley, Kerry Bode, Judith Sharp, Rosie Wragg, Beverley Jackson, Samuel Craven, Duncan Spalding, Matyas Fehervari, Madhava Pai, Laith Alghazawi, Anjola Onifade, Parthi Srinivasan, Julliette Ribaud, Ashitha Nair, Michael Mariathasan, Niamh Grayson, Brian R Davidson, Stephanos Pericleous, Krishna Patel, Conrad Shaw, Nolitha Morare, Mohamad Khish Zaban, Ricky H Bhogal, Joseph Doyle, Vasileios K Mavroeidis, Daniel Croagh, Ismael Dominguez, Alan Guerrero, Andre Moguel, Carlos Chan, Rohan Thakkar, Michael Jones, Edward Buckley, Nasreen Akter, Kyle Treherne, Dhanny Gomez, Gregory Gordon, Michael A Silva, Daniel Hughes, Tomas Urbonas, Pierfrancesco Lapolla, Andrea Mingoli, Gioia Brachini, Roberto Caronna, Piero Chirletti, Alberto Porcu, Teresa Perra, Nehal S Shah, Nurul Nadhirah Abd Kahar, Thomas Hall, Nabeegh Nadeem, Zaed Z R Hamady, Shoura Karar, Ali Arshad, Bilal Al-Sarrieh, Adam Yarwood, Mohammed Hammoda, Alejandro Serrablo, Maria Artigas, Sandra Paterna-López

Supplementary Materials - Index

Supplementary Figures and Tables

Table S1 *pag. 4*

Table S2 *pag. 4*

Table S3 *pag. 5*

Figure S1 *pag. 6*

Supplementary Figures and Tables

Table S1. Pattern of recurrence considering the type of resection and the margin resection status

Type of recurrence	PD + R0 (n=133) n, (%)	PD + R1 (n=29) n, (%)	PDVR + R0 (n=47) n, (%)	PDVR + R1 (n=34) n, (%)	*p*
**Local**	42 (31.6)	12 (41.4)	20 (42.6)	15 (44.1)	0.351
**Systemic**	45 (33.8)	15 (51.7)	20 (42.6)	20 (58.8)	**0.034[#]**

PD: pancreatoduodenectomy; PDVR: pancreatoduodenectomy with concomitant venous resection.
[#] PD+R1 vs PDVR+R0: *p*=0.436; PDVR+R0 vs PDVR+R1: *p*=0.148

Table S2. Survival analysis for factors affecting patients' overall survival using a Cox proportional hazard model (after propensity-score matching)

	Univariable		Multivariable		
**Characteristics**	**Median survival (months)**	***p***	**HR**	**95% CI**	***p***
**Age ≥ 55**	27	0.641			
**Male sex**	27	0.877			
**Diabetes mellitus**	30	0.626			
**Previous malignancies**	26	0.471			
**Respiratory disease**	26	0.815			
**Cardiovascular disease**	28	0.625			
**Neoadjuvant therapy**	24	0.396			
**Biliary stenting** (n=434)	47	0.156	0.682	0.339-1.371	0.283
**Albumin < 35 g/l**	24	0.406			
**Neutrophiles > 7.5 x 10^9/l**	27	0.887			
**Lymphocytes > 4 x 10^9/l**	29	0.875			
**Serum Bilirubin > 17 µmol/l**	23	0.096	1.256	0.911-1.733	0.165
**ASA > 2**	22	0.937			
**Operation technique**		0.862			
PPPD	28				
Whipple	24				
**Venous resection**	23	**0.023**	1.409	0.987-2.012	0.059
**pT stage (AJCC 7th Edition)**		**<0.001**	1.295	0.855-1.960	0.222
T1+2	51				
T3	24				
**pN stage (AJCC 7th Edition)**		**<0.001**	2.088	1.442-3.025	**<0.001**
N0	54				
N1	21				
**Grading (n=400)**		**0.015**	n.a.	n.a.	n.a.
G1	45				
G2	30				
G3	20				
**R1 within SMV-groove**	19	**0.006**	1.149	0.787-1.678	0.472
**Adjuvant therapy**	27	0.751			
**CR-POPF**	13	0.580			
**CR-DGE**	34	0.774			
**CR-POBL**	20	0.360			
**CR-POGL**	30	0.819			
**CR-PPH**	24	0.733			
**PV Thrombosis**	23	0.190	0.649	0.196-2.148	0.479
**Re-Laparotomy after 30 days**	9	**<0.001**	4.359	1.970-9.645	**<0.001**

*Median value (range),
AJCC: American Joint Committee on Cancer; ASA: American Society of Anesthesiologists; CR: clinically relevant; DGE: delayed gastric emptying; ICU: intensive care unit; POBL: post-operative biliary leakage; POGL: post-operative gastrointestinal leakage; POPF: post-operative pancreatic fistula; PPH: post-pancreatectomy hemorrhage; ; PPPD: pylorus preserving pancreatoduodenectomy; PV: portal vein; SMV: superior mesenteric vein; VR: venous resection.

Table S3. Survival analysis for factors affecting patients' disease-free survival using a Cox proportional hazard model (after propensity-score matching)

	Univariable		Multivariable		
**Characteristics**	**Median survival (months)**	***p***	***HR***	**95% CI**	***p***
**Age ≥ 55**	21	0.500			
**Male sex**	19	0.381			
**Diabetes mellitus**	25	0.639			
**Previous malignancies**	18	0.456			
**Respiratory disease**	21	0.349			
**Cardiovascular disease**	20	0.496			
**Neoadjuvant therapy**	14	0.354			
**Biliary stenting** (n=434)	Not reached	0.369			
**Albumin < 35 g/l**	17	0.287			
**Neutrophiles > 7.5 x 10^9/l**	21	0.145	1.118	0.708-1.993	0.514
**Lymphocytes > 4 x 10^9/l**	21	0.134	1.093	0.550-2.170	0.799
**Serum Bilirubin > 17 µmol/l**	19	0.081	1.322	0.924-1.892	0.127
**ASA > 2**	17	0.399			
**Operation technique**		0.160	0.805	0.565-1.146	0.228
PPPD	17				
Whipple	21				
**Venous resection**	17	**0.043**	1.392	0.946-2.047	0.093
**pT stage (AJCC 7th Edition)**		**<0.001**	1.514	0.934-.2.453	0.092
T1+2	Not reached				
T3	17				
**pN stage (AJCC 7th Edition)**		**<0.001**	1.524	1.012-2.295	**0.044**
N0	46				
N1	16				
**Grading (n=400)**		0.088	n.a.	n.a.	n.a.
G1	26				
G2	21				
G3	15				
**R1 within SMV-groove**	15	**0.010**	1.129	0.741-1.719	0.573
**Adjuvant therapy**	18	**0.008**	1.303	0.794-2.137	0.295
**CR-POPF**	31	0.369			
**CR-DGE**	31	0.668			
**CR-POBL**	9	0.082	2.913	0.692-12.270	0.145
**CR-POGL**	28	0.865			
**CR-PPH**	21	0.894			
**PV Thrombosis**	Not reached	0.476			
**Re-Laparotomy after 30 days**	8	**0.019**	3.785	1.496-9.579	**0.005**

*Median value (range),
AJCC: American Joint Committee on Cancer; ASA: American Society of Anesthesiologists; CR: clinically relevant; DGE: delayed gastric emptying; ICU: intensive care unit; POBL: post-operative biliary leakage; POGL: post-operative gastrointestinal leakage; POPF: post-operative pancreatic fistula; PPH: post-pancreatectomy hemorrhage; ; PPPD: pylorus preserving pancreatoduodenectomy; PV: portal vein; VR: venous resection.

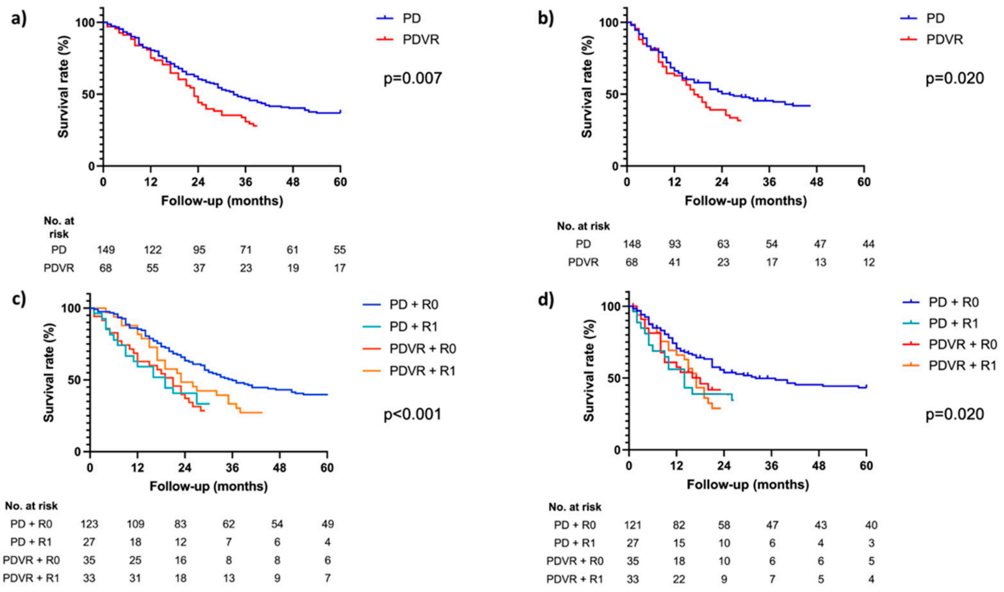

Figure S1. Kaplan-Meier survival curves concerning a) OS and b) DFS after PSM by considering only patients undergoing upfront surgery; c) OS and d) DFS considering the combination of the type of resection (PD vs PDVR) together with the resection margin status (R0 vs R1) by considering only patients undergoing upfront surgery.
